# Inhibition of HIF1A-AS1 promoted starvation-induced hepatocellular carcinoma cell apoptosis by reducing HIF-1α/mTOR-mediated autophagy

**DOI:** 10.1186/s12957-020-01884-x

**Published:** 2020-05-30

**Authors:** Fenfen Hong, Yu Gao, Yang Li, Linfeng Zheng, Feng Xu, Xianpeng Li

**Affiliations:** 1 Division of Gastroenterology and Hepatology, Yinzhou Hospital Affiliated to Medical School of Ningbo University, Ningbo, Zhejiang, 315000 China; 2 Hongkou Branch of Changhai Hospital, Naval Medical University, Shanghai, 200433 China; 3 Department of Cardiovascular Surgery, Changhai Hospital, Naval Medical University, Shanghai, 200433 China; 4 Department of Radiology, Shanghai General Hospital, Shanghai Jiao Tong University, Shanghai, 200080 China; 5 Ningbo, China

**Keywords:** Hepatocellular carcinoma cells, Apoptosis, Starvation, HIF1A-AS1, Autophagy

## Abstract

**Background:**

Hepatocellular carcinoma (HCC) is still a major health burden in China considering its high incidence and mortality. Long non-coding RNAs (lncRNAs) were found playing vital roles in tumor progression, suggesting a new way of diagnosis and prognosis prediction, or treatment of HCC. This study was designed to investigate the role of HIF1A-AS1 during the progression of HCC and to explore its related mechanisms.

**Methods:**

The expression of HIF1A-AS1 was detected in 50 paired carcinoma tissues and adjacent normal tissues by quantitative real-time PCR assay. HCC cell apoptosis was induced by nutrient-deficient culture medium and detected by Cell Counting Kit-8 and flow cytometer assays. HIF1A-AS1 inhibition in HCC cells was accomplished by small interfering RNA transfection.

**Results:**

HIF1A-AS1 was overexpressed in HCC tissues and was associated with tumor size, TNM stage, and lymph node metastasis. Compared with the low HIF1A-AS1 group, the high HIF1A-AS1 group had a shorter overall survival and a worse disease-free survival. HIF1A-AS1 expression was significantly higher in HCC cell lines (7721 and Huh7) than that in normal hepatocyte cell line L02 under normal culture condition. However, under nutrient-deficient condition, HIF1A-AS1 expression was significantly increased in both HCC and normal hepatocyte cell lines and was increased with the prolongation of nutrient-free culture. Inhibition of HIF1A-AS1 promoted starvation-induced HCC cell apoptosis. Furthermore, inhibition of HIF1A-AS1 could also reduce starvation-induced HCC cell autophagy. The expression of HIF-1α and phosphorylated mTOR was significantly decreased in HCC cells after HIF1A-AS1 inhibition.

**Conclusions:**

HIF1A-AS1, overexpressed in HCC and associated with HCC prognosis, could regulate starvation-induced HCC cell apoptosis by reducing HIF-1α/mTOR-mediated autophagy, promoting HCC cell progression.

## Background

Hepatocellular carcinoma (HCC) is the most common primary liver cancer in the world, and it is one of the most common causes of tumor-related deaths due to the high incidence of tumor recurrence and metastasis worldwide [1, 2]. Although interventional therapy, liver transplantation, chemotherapy, and surgery are available to treat HCC, the 5-year survival rate remains unsatisfactory [3, 4]. In general, the process of HCC involves multiple steps, including a large number of genetic or epigenetic changes, which ultimately lead to the transformation of hepatocellular malignancies [5, 6]. Therefore, in order to improve the diagnosis and management strategies for HCC, it is urgent to find new hepatocellular carcinoma biomarkers and to understand the molecular mechanism of HCC in more detail.

Autophagy is a highly conserved and widespread mechanism of lysosome-dependent self-digestion and intracellular recycling in the evolutionary process, which is essential for the maintenance of cellular homeostasis [7]. The self-protection mechanism of autophagy occurs in unfavorable environment, which is closely related to the occurrence and development of tumor [8]. For instance, autophagy can affect the progression of HCC by regulating the expression of other factors or signaling pathways, such as RAC1, NF-κB, and PI3K pathways [9–11]. However, the specific molecular mechanism between HCC and autophagy remains unclear.

Long non-coding RNAs (lncRNAs) are commonly defined as the > 200-nt transcript without protein-coding potential. lncRNAs have been identified as one of the most important regulatory factors in medical research in recent years, which play a complex and precise regulatory function in the development of organism and disease [12]. Recently, it has been reported that several lncRNAs are frequently regulated in HCC, including HOTAIR, MALAT1, UCA1, HULC, DBH-AS1, and PTV1 [13–18]. Some of these lncRNAs include HOTAIR, HULC, PTV1, and MALAT1, which are also involved in the regulation of autophagy in hepatoma cells [19–22]. lncRNA HIF1A-AS1 is located on the antisense strand of hypoxia inducible factor 1α (HIF-1α) of human chromosome 14, and the length of mature body is 652 nt. Zhang et al. found that the activation of hepatic stellate cells mediated by TET3 may be mediated by regulating the HIF1A-AS1 expression [23]. The expression of HIF1A-AS1 is closely related to the proliferation and apoptosis of hepatic stellate cells [24]. However, the specific downstream regulation mechanism of HIF1A-AS1 has not been reported.

In our study, we aimed to identify the relationship between HIF1A-AS1 expression and clinical characteristic of HCC and to explore the role and mechanism of HIF1A-AS1 on nutrient-deficient induced HCC cell apoptosis and autophagy. The results would bring potential research directions for HCC treatment in future.

## Methods

### Patients and specimens

This study was carried out in accordance to the principles of the Declaration of Helsinki and approved by the Medical Ethics Committee in Ningbo University Medical College. All clinical HCC tissues and matched adjacent normal tissues were obtained from Yinzhou Hospital between 2015 and 2017. Written informed consent was obtained from all the participants prior to enrollment. All patients recruited in this study were diagnosed with HCC based on histopathological evaluation and did not receive any chemotherapy or radiotherapy before surgical operation. All collected tissues were immediately stored at liquid nitrogen until further analysis. TNM staging of HCC samples was performed according to the 7th edition AJCC/UICC TNM staging systems. Clinicopathological characteristic analyses of all the specimens are provided in Table 1.

**Table 1:** Correlation between HIF1A-AS1 levels and clinical features of HCC patients

Clinical features	*n*	HIF1A-AS1 expression		*P*
		High (*n* = 25)	Low (*n* = 25)	
Age				
≤ 50	14	8	6	0.529
> 50	36	17	19	
Gender				
Female	19	9	10	0.771
Male	31	16	15	
Tumor size				
≤ 5cm	25	7	15	0.023*
> 5cm	25	18	10	
TNM stage				
I–II	25	8	16	0.024*
III–IV	25	17	9	
Lymph node metastasis				
Yes	25	15	8	0.047*
No	25	10	17	

*P < 0.05

### Cell lines and culture

The human HCC cell lines (7721 and Huh7) were obtained from the Chinese Academy of Sciences, and the normal hepatocyte cell line (L02) was presented by the Department of General Surgery of Changhai Hospital. Cells were cultured in Dulbecco’s modified Eagle’s medium (Gibco) containing 10% fetal bovine serum (Gibco) in a humidified atmosphere of 5% CO_2_ at 37 °C. The nutrient-deficient induction was established by Earle’s Balanced Salt Solution (EBSS, Gibco).

### RNA extraction and quantitative real-time PCR

Total RNA was extracted from tissue and cell samples using TRIzol reagent (Invitrogen) according to the manufacturer’s protocol. Reverse transcription was performed using PrimeScript RT Reagent Kit (TaKaRa). The diluted cDNAs were amplified using SYBR Premix Ex Taq (TaKaRa). Three independent biological replicates were set at least for the cell experiments. β-Actin was used as a loading control. The sequences of the primers were listed in supplemental Table S1.

### Western blot analysis

Total cell lysates were subjected to 10% SDS-PAGE, and the proteins were transferred to nitrocellulose filter membranes, followed by blocking for 1 h in 5% non-fat dry milk. The membranes were incubated with primary antibodies (LC3, 1:1000 dilutions, ab51520 from Abcam; BECN1, 1:1000 dilutions, ab62557 from Abcam; HIF-1α, 1:500 dilutions, BM4083 from Boster; P-mTOR and mTOR, 1:1000 dilutions, ab32028 and ab2732 from Abcam; β-actin, 1:2000 dilutions, BM0627 from Boster) at 4 °C overnight and then with secondary antibodies (HRP-conjugated anti-mouse and anti-rabbit secondary antibodies, 1:5000 dilutions, BA1051 and BM2006 from Boster) at room temperature for 1 h. Proteins were visualized by ECL Plus Western Blotting Substrate (Thermo Scientific) on ChemiDoc MP system (Bio-Rad). β-Actin was used as a gel loading control.

### Small interfering RNA and transient transfection

siRNA targeting HIF1A-AS1 (5′-GUCAAUUGGUUGAUCACCCG-3′, si-HIF1A-AS1) and scrambled control (5′-UUCUCCGAACGUGUCACGUTT-3′, si-NC) were designed and synthesized by Shanghai GenePharma company. When the confluence of cells reached to 70–80%, siRNAs were transfected at a final concentration of 100 nmol/L with Lipofectamine 2000 (Invitrogen) according to the manufacturer’s protocol. The non-off-target effects were confirmed by an additional siRNA-targeted GAPDH (supplemental Figure S1). Knockdown efficiency of the siRNA was determined by qRT-PCR.

### Cell viability analysis

The HCC cell vitality was identified by Cell Counting Kit-8 assay (CCK-8, Beyotime). Briefly, HCC cells were seeded in a 96-well plate (6 wells per group) and incubated overnight, followed by siRNA transfection and nutrient-deficient induction for 24 h. After adding 10 μL CCK-8 solution, the relative growth vitality was detected on a microplate reader (BioTek) according to the manual.

### Cell apoptosis analysis

The cell apoptosis was determined using Annexin V-fluorescein isothiocyanate (FITC)/propidium iodide (PI) double staining (BD Biosciences). After treatment for 48 h, cells were harvested and resuspended in 200 μL Annexin-binding buffer. Then, the cells were incubated with 10 μL Annexin V-FITC and 5 μL PI for 30 min in the dark. The stained cells were examined by a FACScan flow cytometer (BD Biosciences).

### Statistical analysis

The statistical analysis was carried out by SPSS 22.0 software. The qualitative data was analyzed by chi-square test or Fisher’s exact test when necessary. The quantitative data were expressed as the means ± standard deviations and analyzed by *t* test for 2 groups and one-way ANOVA test for multiple groups. The survival curves were analyzed by the Kaplan-Meier test. A *P* value less than 0.05 was considered statistically significant.

## Results

### The elevated HIF1A-AS1 levels were directly proportional to HCC prognosis

**Fig. 1:**
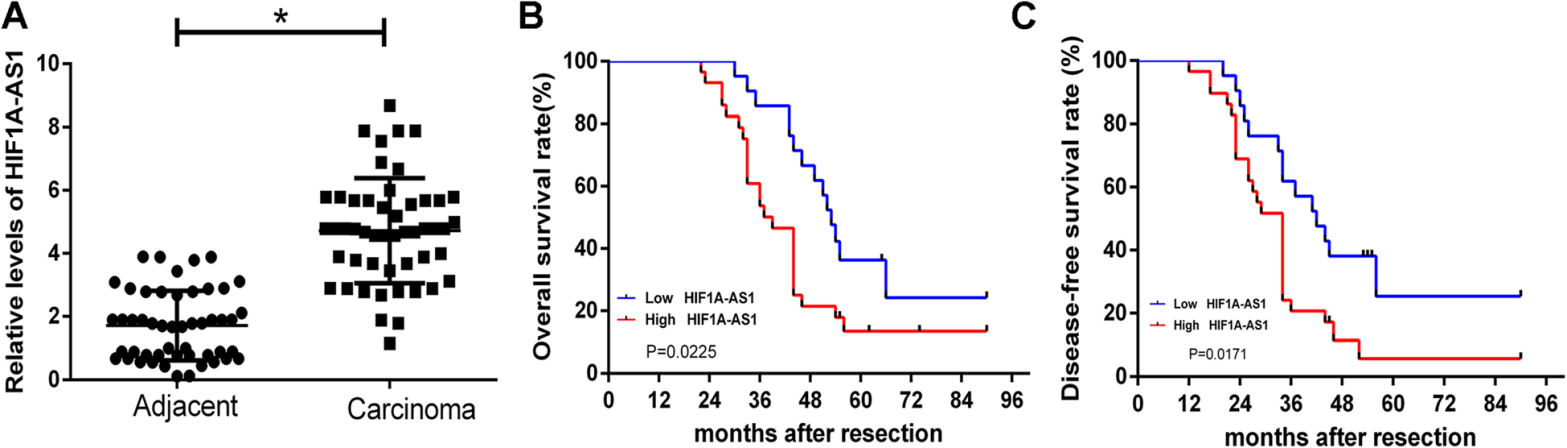
The elevated HIF1A-AS1 levels were directly proportional to HCC prognosis. **a** The relative expression of HIF1A-AS1 was detected in 50 pairs of HCC specimens (carcinoma group) and corresponding adjacent tissues (adjacent group) by qRT-PCR assay and calculated by the 2^-△△CT^ method. HIF1A-AS1 expression was obviously higher in the carcinoma group than that in the adjacent group. *P < 0.05. **b** The Kaplan-Meier overall survival curve for HCC patients with high and low HIF1A-AS1 levels. HCC patients with high HIF1A-AS1 level had significantly shorter overall survival than those with low HIF1A-AS1 level (*P* = 0.0225). **c** The Kaplan-Meier disease-free survival curve for HCC patients. HCC patients with high HIF1A-AS1 level had significantly worse disease-free survival than those with low HIF1A-AS1 level (*P* = 0.0171). The disease-free survival curve was created based on the survival time of HCC patients who did not recur after treatment

We first detected the expression of HIF1A-AS1 in 50 pairs of HCC specimens and corresponding adjacent normal tissues by qRT-PCR. The results showed that HIF1A-AS1 expression was obviously upregulated in HCC specimens when compared with that in matched normal tissues (*P* < 0.01, Fig. 1a). Additionally, we analyzed the correlation between HIF1A-AS1 expression and clinicopathological characteristics in HCC patients, which were divided into the high HIF1A-AS1 group (*n* = 25) and low HIF1A-AS1 group (*n* = 25) with the median value of HIF1A-AS1 expression as a cutoff point. Statistical analysis revealed that high level of HIF1A-AS1 was significantly correlated with tumor size (*P* = 0.023), TNM stage (*P* = 0.024), and lymph node metastasis (*P* = 0.047, Table 1). There was no significant correlation between HIF1A-AS1 expression and other clinicopathological features including age and gender (*P* > 0.05). Furthermore, patients in the high HIF1A-AS1 group had a shorter overall survival (*P* = 0.0225, Fig. 1b) and a worse disease-free survival (*P* = 0.017, Fig. 1c) than the low HIF1A-AS1 group. Together, these results demonstrated that HIF1A-AS1 expression might be associated with the HCC progression and prognosis.

### HIF1A-AS1 expression was increased in HCC cells exposed to starvation

**Fig. 2:**
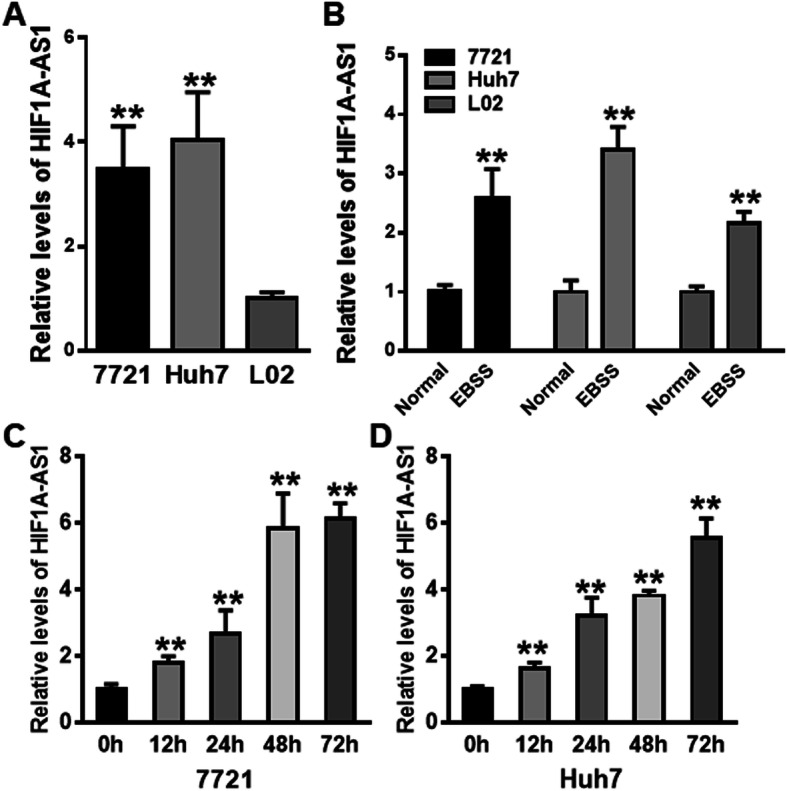
HIF1A-AS1 expression was increased in HCC cells exposed to starvation. **a** The relative expression of HIF1A-AS1 was detected in HCC cell lines (7721 and Huh7) and normal hepatocyte cell line (L02) by qRT-PCR. The HIF1A-AS1 expression in L02 cell line was set as reference, and the relative expression of HIF1A-AS1 in 7721 and Huh7 HCC cell lines was calculated compared with that in L02 cell line. **P < 0.01 vs. L02 group. **b** The expression changes of HIF1A-AS1 in 7721, Huh7, and L02 cell lines under normal and EBSS culture conditions. **P < 0.01 vs. normal group. **c**, **d** The expression changes of HIF1A-AS1 in 7721 and Huh7 HCC cell lines at different starvation time. **P < 0.01 vs. 0 h group

To clarify the potential function of HIF1A-AS1 on HCC development, we first detected HIF1A-AS1 expression levels in different HCC cells. On normal culture condition, the expression level of HIF1A-AS1 was significantly higher in HCC cell lines (7721 and Huh7) than that in normal hepatocyte cell line L02 (Fig. 2a). Under nutrient-deficient condition, HIF1A-AS1 expression was significantly increased in both HCC and normal hepatocyte cell lines (Fig. 2b). Furthermore, we analyzed the relationship between HIF1A-AS1 expression and the nutrient-deficient time in 2 HCC cell lines. qRT-PCR assay confirmed that the expression of HIF1A-AS1 was increased with the prolongation of nutrient-free culture in both 7721 and Huh7 cell lines (Fig. 2c, d). These results indicated that the HIF1A-AS1 might be correlated with nutrient-deficient induced HCC cell apoptosis or autophagy.

Inhibition of HIF1A-AS1 promoted starvation-induced HCC cell apoptosis

**Fig. 3:**
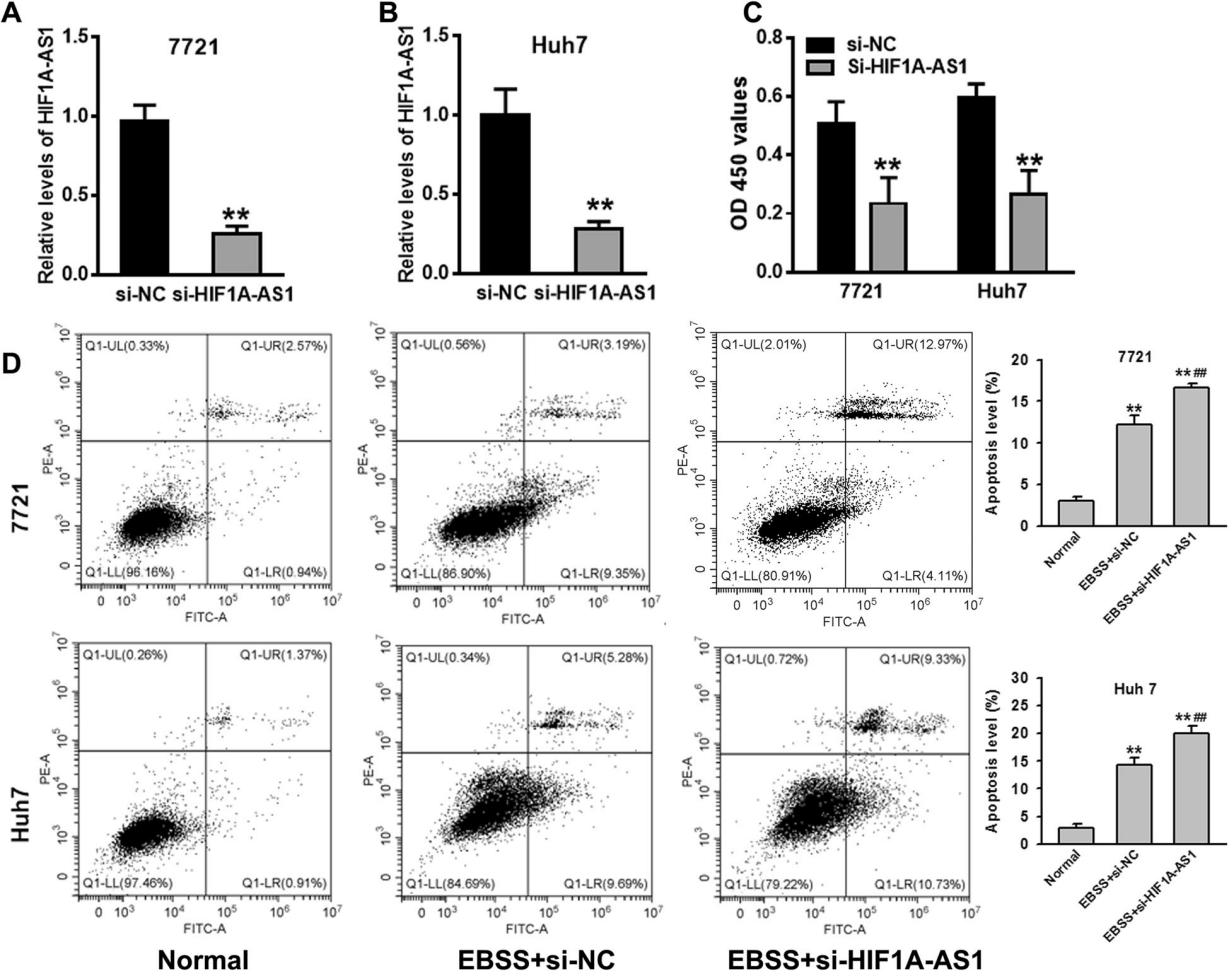
Inhibition of HIF1A-AS1 promoted starvation-induced HCC cell apoptosis. **a**, **b** The inhibition efficiency of siRNA targeting HIF1A-AS1 in 7721 and Huh7 HCC cell lines. qRT-PCR assay confirmed that the expression of HIF1A-AS1 was significantly decreased in 7721 and Huh7 HCC cell lines transfecting si-HIF1A-AS1. **P < 0.01 vs. si-NC group. **c** The role of HIF1A-AS1 on HCC cell growth viability under nutrient-deficient condition. CCK-8 assay confirmed that inhibition of HIF1A-AS1 depressed HCC cell growth. **P < 0.01 vs. si-NC group. **d** The role of HIF1A-AS1 on starvation-induced HCC cell apoptosis. Flow cytometry assay confirmed that inhibition of HIF1A-AS1 could enhance HCC cell apoptosis

In order to further explore the role of HIF1A-AS1 on nutrient-deficient induced HCC cell behaviors, siRNAs targeting HIF1A-AS1 (si-HIF1A-AS1) and negative control (si-NC) were synthesized and transfected into HCC cells, respectively. The inhibition efficiency of si-HIF1A-AS1 was verified by qRT-PCR assay. si-HIF1A-AS1 transfection could significantly reduce the HIF1A-AS1 level in 7721 and Huh7 HCC cell lines (Fig. 3a, b). The result from CCK-8 assay showed that the viability of HCC cells transfected with si-HIF1A-AS1 was significantly lower than that of the si-NC group under nutrient-deficient condition (Fig. 3c). Flow cytometry assay confirmed that inhibition of HIF1A-AS1 could enhance nutrient-deficient induced HCC cell apoptosis (Fig. 3d).

### Inhibition of HIF1A-AS1 reduced starvation-induced HCC cell autophagy

**Fig. 4:**
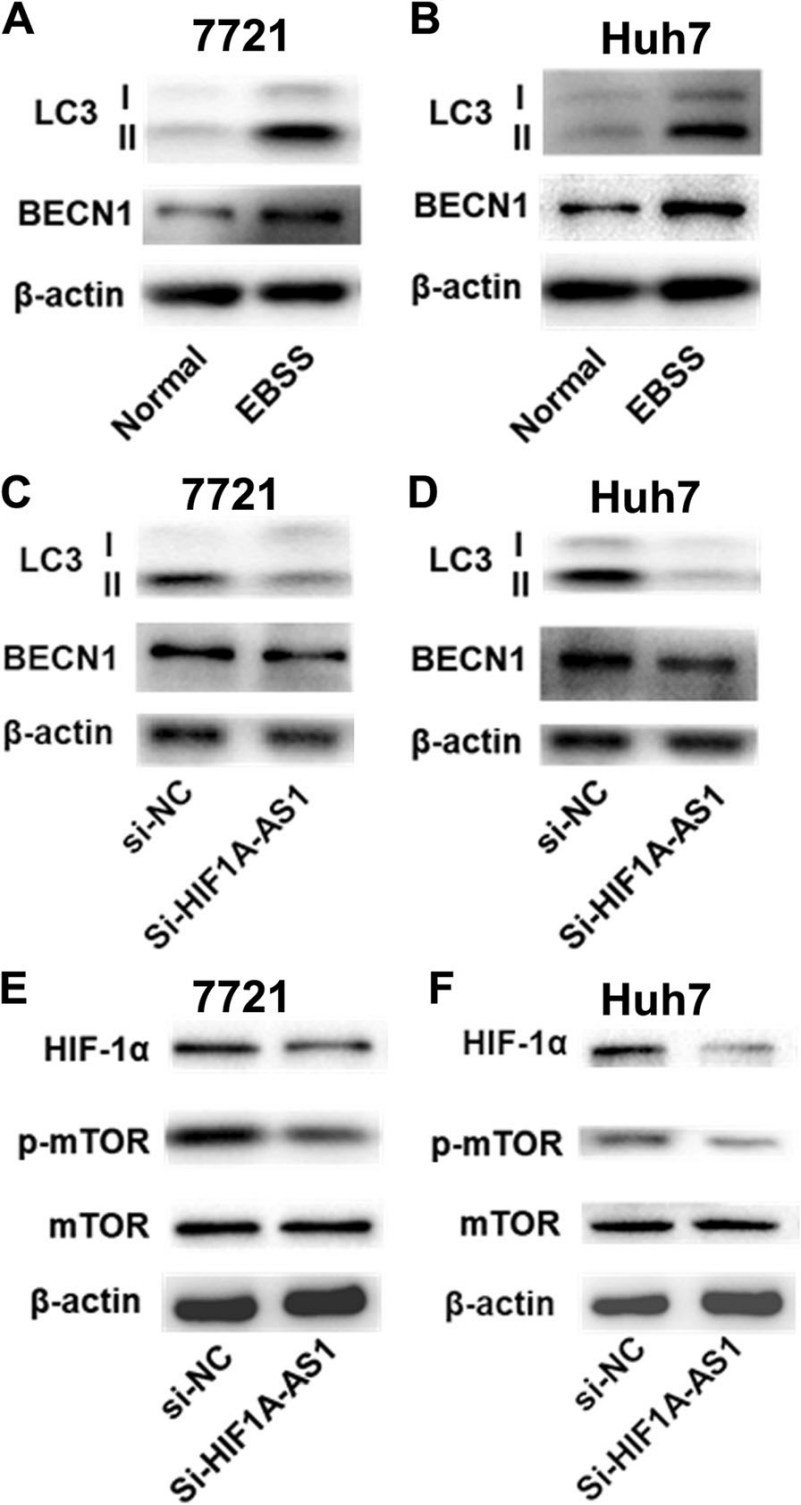
Inhibition of HIF1A-AS1 reduced starvation-induced HCC cell autophagy. Three biological replicates were set for each group in the experiments. **a**, **b** The starvation-induced autophagic activity of HCC cell lines was detected by western blot assay. The expression of BECN1 and the conversion of LC3I to LC3II were significantly increased in 7721 and Huh7 HCC cell lines after 12-h EBSS culture. **c**, **d** The role of HIF1A-AS1 on starvation-induced HCC cell autophagic activity. The expression of BECN1 and the conversion of LC3I to LC3II were significantly decreased in 7721 and Huh7 HCC cell lines transfected with si-HIF1A-AS1 under starvation condition. **e**, **f** The role of HIF1A-AS1 on HIF-1α and mTOR pathway in 7721 and Huh7 HCC cell lines. The expression of HIF-1α and phosphorylated mTOR was significantly decreased after HIF1A-AS1 inhibition

Autophagy is an important mechanism for cells to survive and resist apoptosis in extreme environments, such as nutrient deficiency and hypoxia. Western blot assay showed that the expression of autophagic marker (BECN1 and LC3) was significantly increased in 7721 and Huh7 cell lines after 12-h nutrient-deficient culture (Fig. 4a, b and supplemental Figure S2A-2B). Subsequently, we explored the role of HIF1A-AS1 on HCC cell autophagy induced by nutrient deficiency. After 12-h nutrient-deficient induction, the increased expression of BECN1 and the conversion of LC3I to LC3II (LC3 II/I) were significantly reduced in both 7721 and Huh7 cell lines transfected with si-HIF1A-AS1 (Fig. 4c, d and supplemental Figure S2C-2D). Then, we further detected the effect of HIF1A-AS1 on HIF-1α and mTOR expression under normal and nutrient-deficient conditions. The results showed that the expression of HIF-1α and phosphorylated mTOR was significantly reduced in 7721 and Huh7 HCC cell lines after HIF1A-AS1 inhibition (Fig. 4e, f and supplemental Figure S2E-2F). These results suggested that HIF1A-AS1 might regulate starvation-induced HCC cell autophagy through HIF-1α and mTOR pathways.

## Discussion

In the present study, we found that HIF1A-AS1 was highly expressed in HCC tissues and associated with poor HCC prognosis. Inhibition of HIF1A-AS1 could promote starvation-induced HCC cell apoptosis, which might be mediated by HIF-1α pathway-related autophagy. Our results suggested that inhibition of HIF1A-AS1 might be a potential strategy for HCC treatment.

Many studies have shown that lots of differentially expressed lncRNAs are involved in regulation of HCC cell proliferation, migration, and invasion [2, 25]. lncRNA TUG1, highly expressed in HCC cells, could promote proliferation and apoptosis of HCC cells through epigenetic silencing of KLF2 [26]. Wang et al. found that lncRNA PVT1 can stabilize NOP, thereby promoting HCC cell growth and maintaining stem cell-like properties [27]. lncRNA Magic2-AS3 could inhibit HCC progression by targeting the miR-374b-5p/SMG1 pathway [28]. Our work confirmed that inhibition of HIF1A-AS1 could promote starvation-induced HCC cell apoptosis. Based on the limited protein-coding potential of lncRNAs, overexpression or inhibition of lncRNAs would be a more feasible strategy for gene therapy in HCC.

HIF1A-AS1, located in the antisense strand of human HIF-1α gene, was recognized as an oncogene in non-small cell lung cancer and colorectal cancer [29, 30]. In addition, studies have shown that HIF1A-AS1 was involved in the regulation of proliferation of vascular smooth muscle cells, which may be related to the pathogenesis of aneurysms [31–33]. However, the role of HIF1A-AS1 in HCC remains unclear. In our study, the elevated expression of HIF1A-AS1 was associated with tumor size, TNM stage, lymph node metastasis, and prognosis. These pieces of evidence suggested that HIF1A-AS1 played an important role during the progression of HCC.

Autophagic cell death is a process of programmed cell death that is different from apoptosis and does not depend on the caspase pathway. The autophagic activity would be increased in the harsh environments, including nutritional deprivation, peroxidative damage, and DNA damage. During the development of HCC, in order to adapt to nutrient deficiencies and hypoxia, cells would initiate autophagy to protect themselves [34]. Therefore, inhibition of tumor cell autophagy is an important part of anti-tumor drug development. To date, there have been some studies on the relationship between lncRNA and HCC cell autophagy. Yang et al. found that HOTAIR activated autophagy by increasing the expression of ATG3 and ATG7 [19]. Xiong et al. found that HULC triggered protective autophagy by stabilizing SIRT1 [20]. Yuan et al. found that MALAT1 could inhibit HCC cell apoptosis and reduce chemosensitivity by promoting autophagy [21]. In our experiments, it was found that in the starved state of HCC cells, the highly expressed HIF1A-AS1 is involved in autophagy activation and reduces the HCC cell apoptosis.

mTOR is a mammalian target of rapamycin, an atypical serine/threonine protein kinase. As a key regulator of the autophagy initiation phase, inhibition of mTOR complex 1 (MTORC1) in autophagy has been demonstrated [35]. Li et al. found that DCST1-AS1 accelerates proliferation, metastasis, and autophagy of HCC cells through the Akt/mTOR signaling pathway [36]. Zhang et al. found that SOCS5 promotes HCC cell metastasis via PI3K/Akt/mTOR-mediated autophagy pathway [28]. In our study, we found that inhibition of HIF1A-AS1 induced decreased expression of HIF-1α, suggesting that the role of HIF1A-AS1 on HCC cells autophagy might be mediated by HIF-1α/mTOR pathway.

In the present study, the effect of HIF1A-AS1 on HCC cells was accomplished through in vitro experiments. Inhibition of HIF1A-AS1 was proved to promote HCC cell apoptosis by reducing HIF-1α/mTOR-mediated autophagy, but the function of HIF1A-AS1 on HCC development had not been confirmed through in vivo experiments. Currently, most of the in vitro experiments testing cell biological behavior were performed in substrates coated with protein or peptide ligands for integrins. However, the native extracellular matrix (ECM) is highly enriched with glycosaminoglycans and proteoglycans which could adjust cell adhesion and signaling though integrins [37, 38]. It was reported that hyaluronic acid (HA) together with integrin ligands could promote Huh7 cells spreading on very soft substrates [39]. Recently, an ECM microarray screening platform was developed to screen the effects of substrate stiffness and ECM protein composition and their interactions on cell fate, and could be broadly applied to various types of cells for cell phenotype investigation [40]. The development and optimization of biomimetic cell culture substrates would make the in vitro experiments close to the in vivo microenvironment.

## Conclusions

In conclusion, our results suggest that HIF1A-AS1 promotes hepatocarcinogenesis through activation of autophagy via the HIF-1α/mTOR signaling pathway, and reveal that HIF1A-AS1 is involved in a new pathway to regulate HCC progression and provides a potential direction for future HCC treatment strategies.

## Supplementary information

**Additional file 1: Table S1.** The sequences of the primers in qRT-PCR assay. **Figure S1.** The non-off target effects confirmation of siRNAs in HCC cell lines. The siRNA targeting GAPDH (si-GAPDH) was used to test the non-off target effects of si-HIF1A-AS1 in 7721 (left panel) and Huh7 (right panel) cells. The results from qRT-PCR assay showed that si-GAPDH transfection didn’t effect HIF1A-AS1 expression, just only decreased the expression of GAPDH. **P<0.01 vs. si-NC group. **Figure S2.** The quantification and statistical analysis of western blot assay. The relative gray values were evaluated by software Image J. β-actin was applied as loading control.

## Data Availability

The datasets used during the current study are available from the corresponding author on reasonable request.

## Abbreviations

CCK-8: Cell Counting Kit-8
FITC: Fluorescein isothiocyanate
HCC: Hepatocellular carcinoma cells
HIF-1α: Hypoxia inducible factor 1α
lncRNAs: Long non-coding RNAs
PI: Propidium iodide
siRNA: Small interfering RNA
