# Supplementary material for: Inhibition of HIF1A-AS1 promoted starvation-induced hepatocellular carcinoma cell apoptosis by reducing HIF-1α/mTOR-mediated autophagy
Source: World J Surg Oncol. 2020 May 30;18:113. doi: 10.1186/s12957-020-01884-x (PMC7261383; doi:10.1186/s12957-020-01884-x)
Supplement: Supplementary file 1 — Additional file 1: Table S1. The sequences of the primers in qRT-PCR assay. Figure S1. The non-off target effects confirmation of siRNAs in HCC cell lines. The siRNA targeting GAPDH (si-GAPDH) was used to test the non-off target effects of si-HIF1A-AS1 in 7721 (left panel) and Huh7 (right panel) cells. The results from qRT-PCR assay showed that si-GAPDH transfection didn’t effect HIF1A-AS1 expression, just only decreased the expression of GAPDH. **P<0.01 vs. si-NC group. Figure S2. The quantification and statistical analysis of western blot assay. The relative gray values were evaluated by software Image J. β-actin was applied as loading control. [file 12957_2020_1884_MOESM1_ESM.docx]

Supplemental data

Table S1 The sequences of the primers in qRT-PCR assay

| Targets | Forward | Reverse |
| --- | --- | --- |
| HIF1A-AS1 | 5’-ACGCGGAGAAGAGAAGGAAA-3’ | 5’-TACCGAAGAATGGAGCTGGG-3’ |
| β-actin | 5’-TGGCATCCACGAAACTACCT-3’ | 5’-CGTACAGGTCTTTGCGGATG-3’ |
| GAPDH | 5’- ACTAGGCGCTCACTGTTCTC-3’ | 5’-CCATGGTGTCTGAGCGATGT-3’ |

Figure S1 The non-off target effects confirmation of siRNAs in HCC cell lines. The siRNA targeting GAPDH (si-GAPDH) was used to test the non-off target effects of si-HIF1A-AS1 in 7721 (left panel) and Huh7 (right panel) cells. The results from qRT-PCR assay showed that si-GAPDH transfection didn’t effect HIF1A-AS1 expression, just only decreased the expression of GAPDH. **P<0.01 vs. si-NC group.


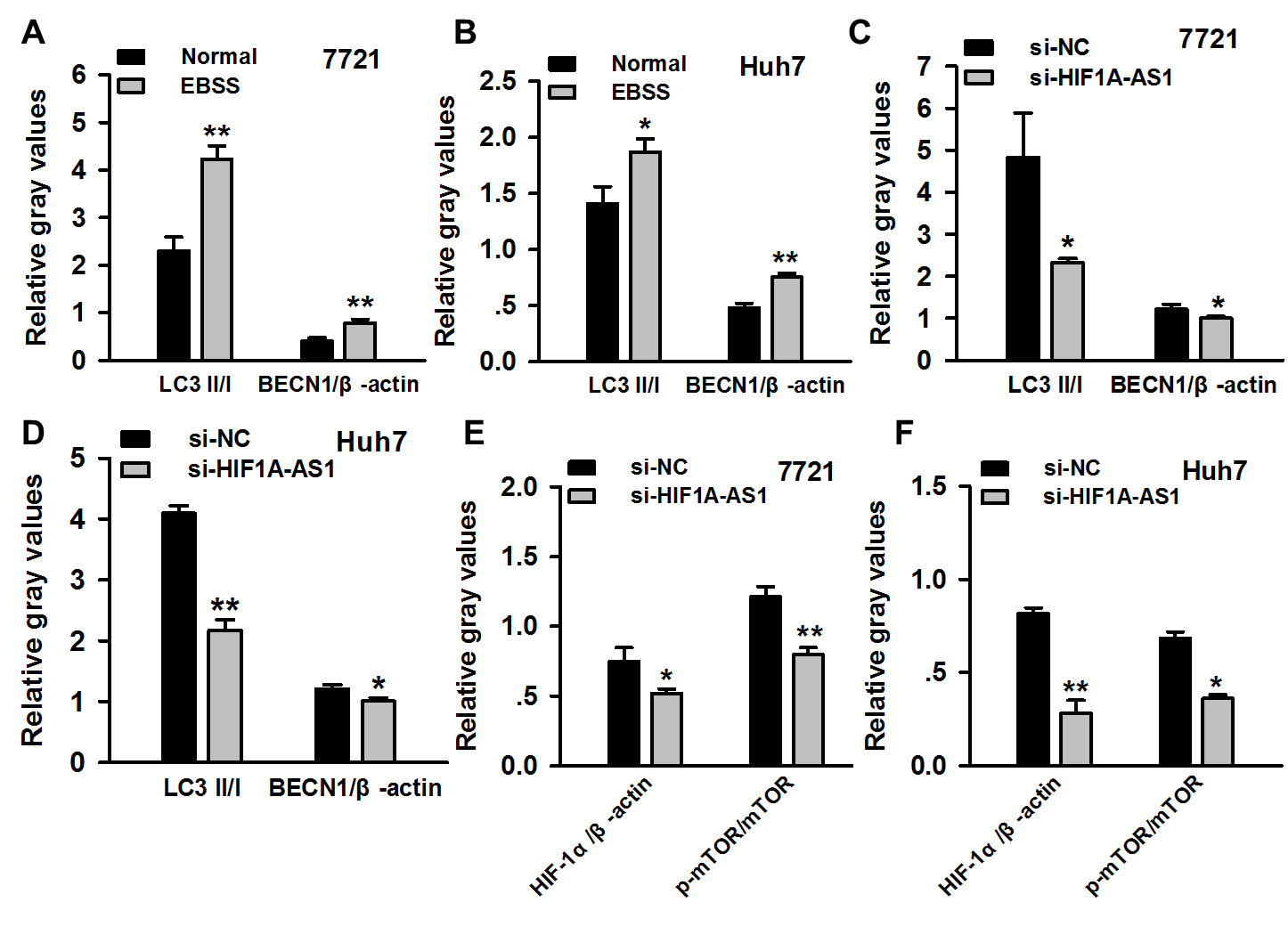


Figure S2 The quantification and statistical analysis of western blot assay. The relative gray values were evaluated by software Image J. β-actin was applied as loading control.
